# Phosphatidylinositol-3-kinase α catalytic subunit gene somatic mutations in bronchopulmonary neuroendocrine tumours

**DOI:** 10.3892/or.2012.2017

**Published:** 2012-09-04

**Authors:** ALESSANDRA CAPODANNO, LAURA BOLDRINI, GRETA ALÌ, SERENA PELLICCIONI, ALFREDO MUSSI, GABRIELLA FONTANINI

**Affiliations:** 1 Department of Surgery, Division of Pathological Anatomy, University of Pisa, I-56124 Pisa, Italy; 2 Department of Cardio-Thoracic Surgery, Division of Thoracic Surgery, University of Pisa, I-56124 Pisa, Italy

**Keywords:** bronchopulmonary neuroendocrine tumours, PI3K/Akt/mTOR pathway, phosphatidylinositol-3-kinase α catalytic subunit mutations, biomarkers

## Abstract

Bronchopulmonary neuroendocrine tumours (BP-NETs) comprise a large spectrum of tumours including typical carcinoids (TCs), atypical carcinoids (ACs), large-cell neuroendocrine carcinomas (LCNECs) and small-cell lung carcinomas (SCLCs) that exhibit considerably different biological aggressiveness and clinical behaviours. The phosphatidylinositol-3-kinase α catalytic subunit (*PIK3CA*) gene is known to be involved in the pathogenesis of several types of human cancers through gene amplification, deletions or somatic missense mutations within the helical and catalytic domains. However, the *PIK3CA* gene status in BP-NETs has yet to be explored. This study aimed to investigate the *PIK3CA* gene status in a large series of BP-NETs by direct gene sequencing and to analyse its correlation with the main clinicopathological parameters. To the best of our knowledge, we demonstrated for the first time a high frequency of somatic missense mutations (23.2%) in the *PIK3CA* gene in a series of 190 BP-NETs, including 75 TCs, 23 ACs, 17 LCNECs and 75 SCLCs. The frequency of the *PIK3CA* gene mutation in the kinase domain was higher (17.9%) than that in the helical domain (5.3%). When the mutational status of the *PIK3CA* gene was compared with the main clinical and pathological characteristics of the BP-NET patients, we found a significant association between *PIK3CA* gene mutations and BP-NET histology (P=0.011). Interestingly, the frequency of *PIK3CA* gene mutations increased with the biological aggressiveness of all BP-NETs, except LCNECs. In conclusion, our results suggest that *PIK3CA* gene mutations may play a key role in tumourigenesis and aggressiveness of BP-NETs. The *PIK3CA* gene may represent a favourable candidate for an effective therapeutic strategy in the treatment of patients with BP-NETs.

## Introduction

Bronchopulmonary neuroendocrine tumours (BP-NETs) comprise approximately 20% of lung cancers and represent a large spectrum of tumours arising from neuroendocrine cells of the bronchopulmonary epithelium. Although they share structural, morphological, immunohistochemical and ultrastructural features, BP-NETs exhibit considerably different biological characteristics and clinical behaviours. Based on their increasing biologic aggressiveness, BP-NETs are separated into four different subgroups: low-grade typical carcinoids (TCs), intermediate-grade atypical carcinoids (ACs) and two high-grade malignancies, large-cell neuroendocrine carcinomas (LCNECs) and small-cell lung carcinomas (SCLCs) (1–3).

TCs and ACs account for ~1–2% of primary lung cancers. The slow-growing TCs exhibit a fairly good prognosis, although ~10–23% of cases when diagnosed metastasize to the regional lymph nodes, with a 5-year overall survival rate ranging from 82 and 100% (2–5). In contrast, ~40–50% of ACs metastasize to the regional lymph nodes when diagnosed, with a 5-year overall survival rate of ~50% (2–5).

SCLCs are the most common BP-NETs and account for 15–20% of invasive lung malignancies, while LCNECs are a very rare neoplasia and represent <1% of lung cancers. LCNECs are currently considered a distinct subtype of non-small cell lung cancer (NSCLC) and are classified as a variant of large-cell carcinomas (LCCs), whose diagnosis may be difficult to establish because of the difficulties in distinguishing LCNECs from poorly differentiated adenocarcinomas, squamous cell carcinomas and basaloid carcinomas. In spite of the several differences between high-grade LCNECs and SCLCs, both progress rapidly, are aggressively metastatic and have a very poor prognosis with a 5-year overall survival rate of 15 to 57% and <5%, respectively (2–5).

The management of BP-NETs is largely dependent on the grade of differentiation (low-to-intermediate grade vs. high-grade) and clinical stage when diagnosed (localized vs. metastatic disease). Surgical resection is the treatment of choice for low-to-intermediate grade and localized tumours, whereas chemotherapy is generally preferred for high-grade and/or disseminated lesions. However, traditional therapies, including radiotherapy and systemic chemotherapy with DNA-damaging cytotoxic agents, are not effective and offer limited benefits to patients with advanced disease (6,7). The lack of effectiveness of traditional agents for BP-NETs has led to an extensive exploration of the molecular profile of these tumours in order to clarify the molecular mechanisms of BP-NET carcinogenesis and progression and to identify new targets for innovative therapies.

The phosphatidylinositol-3-kinase (PI3K)/Akt signalling pathway plays a key role in essential cellular processes, such as cell proliferation, cell growth, apoptosis, metabolism, transcription, protein synthesis, angiogenesis, and tissue invasion, and is involved in the pathogenesis of several types of human cancers (8–11).

Class IA PI3K is a heterodimeric lipid kinase composed of a regulatory subunit (p85α, p85β, p50α, p55α, or p55γ, collectively called p85) and a catalytic subunit (p110α, p110β, or p110δ, collectively called p110). Upon activation by multiple receptor kinase families, including receptor tyrosine kinases and G protein-coupled receptors, PI3K phosphorylates phosphatidylinositol-4,5-bisphosphate (PIP2) to produce phosphatidylinositol-3,4,5-trisphosphate (PIP3), a process that is reversed by the lipid and protein phosphatase PTEN (phosphatase and tensin homologue deleted on chromosome 10). PIP3 acts as a docking site and recruits pleckstrin homology-domain containing proteins to the plasma membrane, such as the serine/threonine kinase Akt and phosphoinositide-dependent kinase 1 (PDK1). Once localized to the plasma membrane, Akt is activated by phosphorylation on threonine 308 in the kinase domain and on serine 473 in the regulatory domain by PDK1 and PDK2, respectively. The active Akt phosphorylates multiple downstream targets namely involved in cell survival, cell cycle progression, cell motility and metabolism (8–11).

Several studies have reported phosphatidylinositol-3-kinase α catalytic subunit (*PIK3CA*) gene amplification, deletions and somatic missense mutations in several types of human cancers, including colorectal, breast and hepatocellular carcinomas where these mutations occur in up to 30% of the tumours examined (12–14).

*PIK3CA* is a 34 kb gene located on chromosome 3q26.3 and consists of 20 exons encoding for the p110α catalytic subunit of PI3K. A number of *PIK3CA* missense mutations are clustered in two *PIK3CA* mutational hotspots and affect conserved regions within the helical (exon 9) and catalytic (exon 20) domains of p110α. The crystal structure of the complex between p110α and p85α has revealed that a number of the cancer-associated *PIK3CA* mutations occur at residues lying at the interfaces between p110α and p85α or between the kinase domain of p110α and other domains within the catalytic subunit. *In vitro* and *in vivo* studies show that these mutations lead to enhanced enzymatic activity, upregulation of the signalling cascade and oncogenic transformation (15–17). Due to the importance of the PI3K/Akt pathway in tumourigenesis and the high frequency of *PIK3CA* gene mutations in human cancers, small PI3K inhibitors are regarded as a promising strategy for cancer treatment.

To date, the mutational status of the *PIK3CA* gene in BP-NETs remains unknown. The aim of this study was to analyse the mutational profile of the *PIK3CA* gene in a large series of BP-NETs and to correlate the *PIK3CA* gene status with the main clinicopathological parameters.

## Materials and methods

### Patient selection and tumour characteristics

One hundred and ninety consecutive BP-NETs were retrospectively collected from patients who had undergone surgery at the Department of Cardio-Thoracic Surgery of the University of Pisa between 2000 and 2009. Patients enrolled in this study did not receive chemotherapy and/or radiotherapy before the surgery.

Histological diagnoses and pathological features were reviewed independently by two pathologists (G.A. and G.F.) and formulated according to the 2004 World Health Organization (WHO) classification. Discrepant diagnoses were re-evaluated jointly and discussed until an agreement was reached. Neuroendocrine differentiation was detected by a positive immunohistochemical staining for chromogranin A, synaptophysin and/or CD56 markers.

The selection of patients did not require approval by the Institutional Ethics Committee since all samples were coded and the names of the patients were not revealed.

### DNA isolation

Genomic DNA was isolated from 10 μm sections of formalin-fixed and paraffin-embedded tissues. Tissue digestion was preceded by xylene treatment to remove paraffin, rehydration through a graded series of alcohol and manual macrodissection of the tumour area to obtain at least 70% of the tumour cells. Then, genomic DNA was extracted using the QIAamp DNA mini kit (Qiagen) according to the manufacturer’s instructions for paraffin-embedded tissues. The DNA quality and quantity were evaluated using a NanoDrop ND-1000 spectrophotometer.

### Mutational analysis of the PIK3CA gene

Mutational analysis of the *PIK3CA* gene (Reference sequence ENSG00000121879) was performed by PCR amplification and direct gene sequencing of the helical and kinase domains of PI3K encoded by exons 9 and 20, respectively.

Primer pairs flanking *PIK3CA* exons 9 and 20 were selected to avoid the frequent cross-amplification of chromosome 22q (a known *PIK3CA* pseudogene) using the software Primer3 (http://frodo.wi.mit.edu/primer3/). The *PIK3CA* gene was amplified for exon 9 with the primers 5′-ATCATCTGTG AATCCAGA-3′ (forward) and 5′-TTAGCACTTACCTGTG AC-3′ (reverse) and for the exon 20 with the primers 5′-TGAC ATTTGAGCAAAGACC-3′ (forward) and 5′-GTGTGGAAT CCAGAGTGA-3′ (reverse).

PCR amplification was performed in a total volume of 25 μl containing 100 ng of genomic DNA, 12.5 μl of HotStarTaq master mix (Qiagen), 0.5 μl of each primer (20 μM), and water as follows. After an initial denaturation step of 15 min at 95°C, the reaction mixture was run for 40 cycles at 95°C for 30 sec, 50°C for 30 sec, and 72°C for 1 min, followed by a final elongation step at 72°C for 10 min. The efficiency and the quality of the PCR amplification were confirmed by running the PCR products on a 1.5% agarose gel.

The PCR products were subsequently subjected to a purification procedure to remove primers, nucleotides, enzymes and salts using the QIAquick PCR Purification kit (Qiagen).

Cycle sequencing analysis of the purified PCR products was performed with the ABI BigDye Terminator version 3.1 Cycle Sequencing kit (Applied Biosystems) according to the manufacturer’s instructions using the PCR amplification primers for bidirectional sequencing, and the reaction products were purified by ethanol precipitation. Finally, sequence determination was performed using an ABI PRISM 3130 Genetic Analyzer (Applied Biosystems) and data were analysed using the Sequencing Analysis 5.0 Software (Applied Biosystems).

### Statistical analysis

Statistical analyses were performed using the StatView 5.0 Software (Abacus Concepts, Inc., Berkeley, CA). The contingency tables and the Chi-square test were used to analyse the association between the different biological parameters. All the tests were two-tailed and a P-value <0.05 was considered to indicate a statistically significant difference.

## Results

### PI3KCA gene mutation in BP-NETs

To assess the frequency of *PIK3CA* gene mutations in BP-NETs we sequenced genomic DNA isolated from formalin-fixed and paraffin-embedded tissues in a large series of lung neuroendocrine tumours, including 75 low-grade TCs, 23 intermediate-grade ACs, 17 high-grade LCNECs and 75 high-grade SCLCs. Clinical and pathological characteristics of the patients, including patient age, gender, histological type, tumour size, and lymph node status were recorded whenever available and summarized in Table I.

Targeted sequencing of the helical and kinase domains of PI3K revealed that *PIK3CA* gene mutations were present in 44 of the 190 analysed tumours (23.2%). The distribution of mutations between the kinase and the helical domains of PI3K showed that the mutation frequency of the kinase domain (34/190, 17.9%) was approximately three times the mutation frequency of the helical domain (10/190, 5.3%) (Table II).

**Figure 1:**
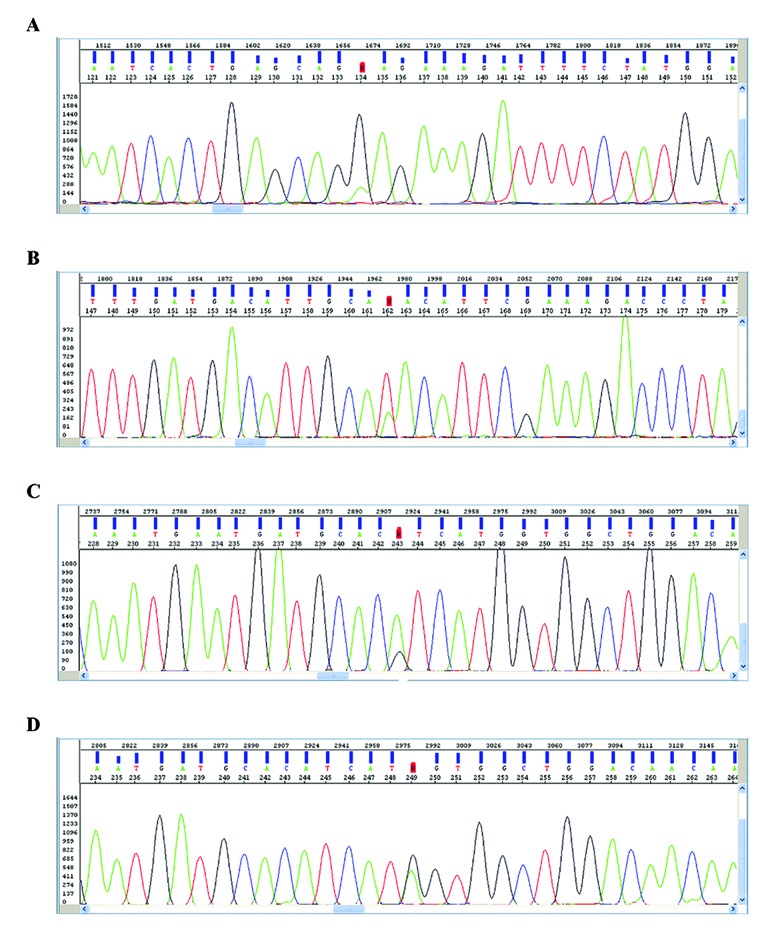
Most frequent mutations of the phosphatidylinositol-3-kinase α catalytic subunit (*PIK3CA*) gene in a series of 190 bronchopulmonary neuroendocrine tumours (BP-NETs). (A) *PIK3CA* helical domain mutation p.E547K (c.1639 G>A). (B) *PIK3CA* kinase domain mutation p.Y1021N (c.3061 T>A). (C) *PIK3CA* kinase domain mutation p.H1047R (c.3140 A>G). (D) *PIK3CA* kinase domain mutation p.G1049S (c.3145 G>A).

All the mutations identified in our series of BP-NETs were single nucleotide substitutions that lead to non-synonymous mutations, including 36 transversions (G↔A or C↔T) and 8 transitions (A↔T, G→T or G→C) (Table II). The most frequent genetic alteration was the E547K mutation in the helical domain accounting for 15.9% (7/44) of the *PIK3CA* gene mutations identified in our study (Table II and Fig. 1A). In the kinase domain we identified 3 different mutational hotspots at codons 1021, 1047, and 1049. The most prevalent genetic anomalies observed in these codons were the mutations Y1021N/F/C (5/44, 11.4%), H1047R (2/44, 4.5%) and G1049S (4/44, 9.1%) (Table II and Fig. 1B-D).

### Correlation between the presence of PIK3CA gene mutations and clinical and pathological parameters

The mutational status of the *PIK3CA* gene was compared with the main clinical and pathological characteristics of the BP-NET patients. The *PIK3CA* gene mutations did not correlate with the clinical and pathological characteristics of the patients, such as age, gender, or lymph node status (Table III). However, statistical analysis showed a significant association between the *PIK3CA* gene mutations in the helical and kinase domains and BP-NET histology (Chi-square test, P=0.011). Our results showed a relatively lower prevalence of *PIK3CA* gene mutations in the low-grade TCs (10/75, 13.3%) compared to the intermediate-grade ACs (9/23, 39.1%) and high-grade SCLCs (23/75, 30.7%). In contrast to the SCLCs, the high-grade LCNECs showed a lower frequency of *PIK3CA* gene mutations (2/17, 11.8%) compared with the other types of BP-NETs (Table III).

## Discussion

BP-NETs comprise a large spectrum of lung cancers ranging from low-grade TCs, to intermediate-grade ACs, to high-grade LCNECs and SCLCs that exhibit considerably different biological aggressiveness and clinical behaviour. At present, the only curative treatment for BP-NETs is radical surgery since traditional therapies are not effective (1,2,5,7).

Recently, successful clinical trials of imatinib, gefitinib/erlotinib and trastuzumab, which are specific for *BCR/ABL* translocations (18), epidermal growth factor receptor (*EGFR*) mutations (19,20) and *HER-2/neu* amplifications (21), respectively, have illustrated the ability to develop drugs that target genetic abnormalities and lead to potential streamlined therapies based on the genomic landscape of an individual’s cancer.

Several studies have shown that the dysregulation of the PI3K/Akt pathway is involved in cancer pathogenesis and prognosis, and *PIK3CA* gene mutations have been reported in several types of human cancers, including colorectal, breast and hepatocellular carcinomas (10–14).

The functional activation of the PI3K/Akt pathway has been investigated in the spectrum of pulmonary or other neuroendocrine tumours only by the indirect evidence of the expression of functionally related molecules, such as PTEN (22), tuberous sclerosis complex (TSC) (23) and mammalian target of rapamycin (mTOR) (24,25). However, the mutational status of the *PIK3CA* gene in BP-NETs remains unknown. This study aimed to explore the mutational profile of the *PIK3CA* gene in a large series of BP-NETs and to determine the correlation of the *PIK3CA* status with the main clinico-pathological parameters.

A number of the somatic mutations involving the *PIK3CA* gene are clustered in exons 9 and 20, which encodes for PI3K helical and kinase domains, respectively (16,17). Previous studies have analysed *PIK3CA* gene mutation in NSCLC and have demonstrated that its frequency is relatively low (3.4–4.3%) compared to that observed in other tumours, such as breast, colon and ovarian cancers. (12–14).

To the best of our knowledge, we demonstrated for the first time a high prevalence of somatic missense mutations (23.2%) in the *PIK3CA* gene in human BP-NETs that is comparable to the mutational frequency of the *PIK3CA* gene in other types of human cancers (12–14,26,27). Moreover, our data are in agreement with the results obtained by Shibata *et al*(28) who have shown three mutations of the *PIK3CA* gene in 13 SCLC cell lines (23%) in an extensive mutational screening. The high frequency of the *PIK3CA* gene mutation in our series of BP-NETs as in several other aggressive human tumours, highlights that somatic mutations of this gene are an important genetic event in BP-NET tumourigenesis and may represent a potentially effective therapeutic target for these types of tumours.

The analysis of the distribution of mutations between the kinase and the helical domains of PI3K in our series of BP-NETs revealed that the frequency of the kinase domain mutations (17.9%) was approximately three times the frequency of mutations in the helical domain (5.3%) according to the findings reported by other authors in different types of human cancers (12–14). We identified four different mutational hotspots: the codon 547 in the helical domain accounting for 15.9% of the *PIK3CA* gene mutations identified in our study and codons 1021, 1047 and 1049 in the kinase domain that together represent 25% of the identified mutations. Several studies have demonstrated a direct connection between mutations in the helical and kinase domain of PI3K and carcinogenesis as well as the prognosis of colorectal and breast cancers (29,30). The probable mechanisms for the oncogenicity of these mutations are the disruption of an inhibitory charge-charge interaction between p110α and the N-terminal SH2 domain of the p85 regulatory subunit and the increased binding affinity of p110α for the negatively charged phosphatidylinositol substrate, as it has been demonstrated by crystallographic and biochemical studies (17,31).

In our current study, a statistically significant correlation was not observed between *PIK3CA* mutations and the main clinical and pathological characteristics of the patients, such as age, gender or lymph node status. However, we found that the frequency of *PIK3CA* gene mutations was significantly associated with BP-NET histology (P=0.011). Interestingly, the prevalence of *PIK3CA* gene mutations increases parallel to the biological aggressiveness of the BP-NETs, since it was relatively lower in the low-grade TCs (13.3%) compared to the intermediate-grade ACs (39.1%) and high-grade SCLCs (30.7%). Notably, the high-grade LCNECs demonstrated an unexpected lower frequency of *PIK3CA* gene mutations (11.8%) compared with the other types of lung neuroendocrine tumours. LCNECs are relatively uncommon tumours, accounting for ~1% of the resected primary lung cancers and represent a controversial entity from both the diagnostic and clinical point of view (2–5). The diagnosis of high-grade neuroendocrine tumours of the lung requires to demonstrate the histopathologic neuroendocrine morphology and the neuroendocrine differentiation using immunohistochemistry or electron microscopy. However, LCNECs are a poorly recognized and underdiagnosed entity as a result of the difficultly in recognizing neuroendocrine morphology and are frequently mistaken for poorly differentiated NSCLCs, ACs and intermediate cell-type SCLCs (5,32,33). Furthermore, although a previous retrospective report demonstrated that the survival rate of patients with surgically resected LCNECs and SCLCs were closely related to each other and inferior to that of patients with TCs and ACs (5), there is no clear-cut evidence concerning the optimal treatment for LCNECs, and therapeutic approaches adopted for SCLCs are not considered effective for patients with LCNEC, thus questioning whether LCNECs are best classified and treated as SCLC or LCC patients (5,32,33).

Our results demonstrated that *PIK3CA* gene mutations were more common in ACs and SCLCs than in LCNECs, suggesting that LCNECs, athough sharing several similarities with SCLCs on morphological, immunohistochemical and molecular grounds, represent a distinct biological entity. In a wide retrospective study, Varlotto *et al,* demonstrated that clinical as well as histopathologic and biological characteristics of LCNECs more closely resemble LCCs than SCLCs (33). The differences in the *PIK3CA* gene mutational status between LCNECs and ACs or SCLC observed in our study may suggest that a different signalling pathway such as the MAPK pathway or other receptor tyrosine kinases (RTKs), but not PI3K, may play a key role in the tumourigenesis of the former type of BP-NETs. In support of this hypothesis, Rossi *et al* demonstrated that LCNECs overexpress several RTKs, including KIT, the platelet-derived growth factor receptors α (PDGFRα) and β (PDGFRβ), and MET in a number of patients, whereas they failed to find a significant expression in other NSCLCs and carcinoids (32,34).

An important implication of this study is the possibility of applying our results in the clinics. *PIK3CA* mutations have been associated with paclitaxel resistance in breast epithelial cells and the PI3K/Akt pathway has been linked with resistance to a number of other cancer therapies (35). In experimental models of human pulmonary carcinoid and SCLC cells, the inhibition of the PI3K/Akt pathway by LY294002 and Tricribine, respectively, significantly reduced cellular growth and neuroendocrine marker expression *in vitro* and increased apoptosis and sensitivity to chemotherapeutic treatments (28,36). Since mutations in the *PIK3CA* gene result in constitutively active PI3K activity, the presence of *PIK3CA* mutations may allow for the selection of patients with a high response rate to novel and targeted strategies of treatment based on the development of compounds designed to target PI3K or more feasibly downstream effectors within the PI3K/Akt signalling pathway.

In conclusion, our results strongly suggest that *PIK3CA* genetic alterations may play an extensive and fundamental role in the tumourigenesis and aggressiveness of BP-NETs and specific-based targeting at the PI3K/Akt signalling pathway may be an effective therapeutic strategy for BP-NET treatment.

## Figures and Tables

**Table I:** Characteristics of the BP-NET patients.

Clinicopathological features	TC	AC	LCNEC	SCLC
Age (years)				
Median (range)	61 (24–82)	58 (23–82)	68 (45–84)	69 (49–83)
Gender^a^	n=75	n=23	n=17	n=75
Male	31 (41.3)	6 (26.0)	13 (76.5)	60 (80.0)
Female	44 (58.7)	17 (74.0)	4 (23.5)	15 (20.0)
Tumour size^a^	n=74	n=23	n=16	n=65
T1 (T1a-T1b)	50 (67.6)	9 (39.1)	4 (25.0)	21 (32.3)
T2 (T2a-T2b)	19 (25.7)	11 (47.8)	7 (43.7)	29 (44.6)
T3	5 (6.7)	3 (13.1)	5 (31.3)	15 (23.1)
Lymph node status^a^	n=65	n=19	n=13	n=56
Negative	62 (95.4)	13 (68.4)	11 (84.6)	34 (60.7)
Positive	3 (4.6)	6 (31.6)	2 (15.4)	22 (39.3)

a Values are shown as n (%).

TC, typical carcinoid; AC, atypical carcinoid; LCNEC, large-cell neuroendocrine carcinoma; SCLC, small-cell lung carcinoma; BP-NET, bronchopulmonary neuroendocrine tumour.

**Table II:** *PIK3CA* gene status in BP-NET patients.

Exon	*PIK3CA* Domain	ID Sample	Histology	Nucleotide substitution	Amino acid substitution	No. of cases
9	Helical	N145	SCLC	c.1573 G>A	p.E525K	1
9	Helical	N56	TC	c.1576 A>G	p.N526D	1
9	Helical	N170	SCLC	c.1624 G>A	p.E542K	1
9	Helical	N20, N46, N50	TC	c.1639 G>A	p.E547K	3
9	Helical	N34	AC	c.1639 G>A	p.E547K	1
9	Helical	N18, N33, N59	SCLC	c.1639 G>A	p.E547K	3
20	Kinase	N188	SCLC	c.2944 G>C	p.E982Q	1
20	Kinase	N101	SCLC	c.2949 G>A	p.M983I	1
20	Kinase	N172	TC	c.2993 T>C	p.F998S	1
20	Kinase	N173	SCLC	c.2998 A>G	p.N1000D	1
20	Kinase	N116	SCLC	c.3007 T>C	p.S1003P	1
20	Kinase	N129	AC	c.3007 T>C	p.S1003P	1
20	Kinase	N97	SCLC	c.3012 G>T	p.M1004I	1
20	Kinase	N8	AC	c.3017 T>C	p.L1006P	1
20	Kinase	N35	SCLC	c.3016 C>T	p.L1006F	1
20	Kinase	N167	SCLC	c.3022 T>C	p.S1008P	1
20	Kinase	N176	AC	c.3022 T>C	p.S1008P	1
20	Kinase	N125	SCLC	c.3032 C>T	p.P1011L	1
20	Kinase	N26	TC	c.3034 G>A	p.E1012K	1
20	Kinase	N95	AC	c.3041 A>G	p.Q1014R	1
20	Kinase	N182	AC	c.3050 A>T	p.D1017V	1
20	Kinase	N67	AC	c.3062 A>G	p.Y1021C	1
20	Kinase	N143	SCLC	c.3062 A>T	p.Y1021F	1
20	Kinase	N88, N157	SCLC	c.3061 T>A	p.Y1021N	2
20	Kinase	N159	TC	c.3061 T>A	p.Y1021N	1
20	Kinase	N17	SCLC	c.3068 G>A	p.R1023Q	1
20	Kinase	N119	AC	c.3068 G>A	p.R1023Q	1
20	Kinase	N138	SCLC	c.3074 C>T	p.T1025I	1
20	Kinase	N115	SCLC	c.3085 G>C	p.D1029H	1
20	Kinase	N14	SCLC	c.3110 A>G	p.E1037G	1
20	Kinase	N11	SCLC	c.3115 T>C	p.F1039L	1
20	Kinase	N152	TC	c.3133 G>A	p.D1045N	1
20	Kinase	N45	SCLC	c.3140 A>G	p.H1047R	1
20	Kinase	N102	LCNEC	c.3140 A>G	p.H1047R	1
20	Kinase	N62, N82	TC	c.3145 G>A	p.G1049S	2
20	Kinase	N114	LCNEC	c.3145 G>A	p.G1049S	1
20	Kinase	N121	SCLC	c.3145 G>A	p.G1049S	1
20	Kinase	N186	AC	c.3155 C>T	p.T1052I	1
Total *PIK3CA* gene mutations^a^						44/190 (23.2%)
Total mutations in the helical domain of the *PIK3CA* gene^a^						10/190 (5.3%)
Total mutations in the kinase domain of the *PIK3CA* gene^a^						34/190 (17.9%)

a Frequencies of mutation are shown as n (%).

TC, typical carcinoid; AC, atypical carcinoid; LCNEC, large-cell neuroendocrine carcinoma; SCLC, small-cell lung carcinoma; BP-NET, bronchopulmonary neuroendocrine tumour.

**Table III:** Correlations between *PIK3CA* gene status and clinicopathological characteristics of patients with BP-NETs.

Clinicopathological features	*PIK3CA* wild-type^a^	*PIK3CA* mutant^a^	P-value^b^
Gender			
Male	84 (76.4)	26 (23.6)	0.992
Female	62 (77.5)	18 (22.5)	
Age (years)			
<66	69 (77.5)	20 (22.5)	0.979
≥66	72 (76.5)	22 (23.5)	
Tumour size			
T1 (T1a-T1b)	67 (79.8)	17 (20.2)	0.972
T2 (T2a-T2b)	50 (75.7)	16 (24.3)	
T3	21 (75.0)	7 (25.0)	
Lymph node status			
Negative	95 (79.2)	25 (20.8)	0.641
Positive	24 (72.7)	9 (27.3)	
Histology			
TC	65 (66.6)	10 (13.3)	
AC	14 (60.9)	9 (39.1)	**0.011**
LCNEC	15 (88.2)	2 (11.8)	
SCLC	52 (69.3)	23 (30.7)	

a Values are shown as n (%);

b P-values were assessed by the Chi-square test and significant P-values are in bold.

TC, typical carcinoid; AC, atypical carcinoid; LCNEC, large-cell neuroendocrine carcinoma; SCLC, small-cell lung carcinoma; BP-NETs, bronchopulmonary neuroendocrine tumours.
